# Stereotyping of medical disability claimants' communication behaviour by physicians: towards more focused education for social insurance physicians

**DOI:** 10.1186/1471-2458-10-666

**Published:** 2010-11-03

**Authors:** HJ van Rijssen, AJM Schellart, M Berkhof, JR Anema, AJ van der Beek

**Affiliations:** 1 Department of Public and Occupational Health, EMGO Institute for Health and Care Research, VU University Medical Center, Amsterdam, The Netherlands; 2 Research Center for Insurance Medicine, collaboration between AMC-UMCG-UWV-VUmc, Amsterdam, The Netherlands

## Abstract

**Background:**

Physicians who hold medical disability assessment interviews (social insurance physicians) are probably influenced by stereotypes of claimants, especially because they have limited time available and they have to make complicated decisions. Because little is known about the influences of stereotyping on assessment interviews, the objectives of this paper were to qualitatively investigate: (1) the content of stereotypes used to classify claimants with regard to the way in which they communicate; (2) the origins of such stereotypes; (3) the advantages and disadvantages of stereotyping in assessment interviews; and (4) how social insurance physicians minimise the undesirable influences of negative stereotyping.

**Methods:**

Data were collected during three focus group meetings with social insurance physicians who hold medical disability assessment interviews with sick-listed employees (i.e. claimants). The participants also completed a questionnaire about demographic characteristics. The data were qualitatively analysed in Atlas.ti in four steps, according to the grounded theory and the principle of constant comparison.

**Results:**

A total of 22 social insurance physicians participated. Based on their responses, a claimant's communication was classified with regard to the degree of respect and acceptance in the physician-claimant relationship, and the degree of dominance. Most of the social insurance physicians reported that they classify claimants in general groups, and use these classifications to adapt their own communication behaviour. Moreover, the social insurance physicians revealed that their stereotypes originate from information in the claimants' files and first impressions. The main advantages of stereotyping were that this provides a framework for the assessment interview, it can save time, and it is interesting to check whether the stereotype is correct. Disadvantages of stereotyping were that the stereotypes often prove incorrect, they do not give the complete picture, and the claimant's behaviour changes constantly. Social insurance physicians try to minimise the undesirable influences of stereotypes by being aware of counter transference, making formal assessments, staying neutral to the best of their ability, and being compassionate.

**Conclusions:**

We concluded that social insurance physicians adapt their communication style to the degree of respect and dominance of claimants in the physician-claimant relationship, but they try to minimise the undesirable influences of stereotypes in assessment interviews. It is recommended that this issue should be addressed in communication skills training.

## Background

Generalising and categorising is necessary to make sense of the complex behaviour of other people. It makes it easier to form coherent impressions of others, and also to understand them. It is, in fact, impossible to start communicating with a stranger without making inferences about that person based on general experiences, and thus stereotyping [1]. The application of general ideas and beliefs about groups of people to individuals is known as stereotyping. Stereotyping increases comprehension, because of its informative value. For example, it enables people to make an educated guess about aspects for which no actual information is available [2,3]. However, stereotyping is also associated with several problems, such as excluding individuals or discriminating them based on prejudices towards groups of people, collective treatment which puts people in an inferior position, and behaviour towards others which leads to stereotype confirmation. Therefore, individual information is generally preferred over stereotyping [2,4,5]. Stereotypes may be applied and discarded during an encounter, but whether or not they are applied in contact with other people depends on many factors, for example on cognitive resources, motivation, and goals [2,6]. Stereotypes may be applied to make communication easier in an initial contact [2].

Studies have indicated that mechanisms of stereotyping can affect a physician's treatment-related decision-making [7], because stereotyping can affect the interpretation of behaviour, symptoms, and diagnosis of patients. Stereotyping can also affect the physician's communication style [7], the physician's behaviour towards the patient [8,9], the patient's motivation and treatment adherence [5,9], and the health care provided [5]. Furthermore, research has convincingly shown that there is no truth in the general belief that physicians are objective and neutral. For example, the demographic characteristics of a patient, such as age, ethnicity, gender, and socioeconomic status, have been found to influence the beliefs and expectations of physicians, especially when complicated assessments, incomplete information, incorrect information, or time-pressure are involved [10,11]. Stereotypes also influence the interpretation of clinical findings, for example because physicians provide inferior care to some groups of patients, due to stereotyping [10].

Social insurance physicians meet their patients (claimants) during the medical disability assessment interview to determine their entitlement to social security benefits. Given the earlier-mentioned research results, these assessments will probably be influenced by the physicians' stereotyping, and especially because one-time contacts are common, claimants will not always be inclined to give correct information, and many claimants have to be assessed in a limited period of time (i.e. approximately one hour per claimant). However, little is known about the mechanisms of the reasoning of physicians during clinical and diagnostic decision-making [12,13]. Moreover, stereotyping is more likely to result when differences in status and power exist between people [9,14], and those differences obviously exist between physicians and their patients during disability assessments. This is especially relevant, because a lot is at stake for the claimants. Yet, very little is known about stereotyping by social insurance physicians, about their handling of information confirming or disconfirming the stereotyping, and about the influences of stereotyping on medical disability assessment interviews.

Previously, our research group has described the conceptualisation of a behavioural model regarding the communication between social insurance physicians and their claimants [15]. This model describes physician-claimant communication from a distance. However, as an actor within the model, one cannot directly observe the other person's intentions and attitudes. Studying the physician-observed determinants of the communication behaviour of claimants, will increase insight into how physicians evaluate claimants and communication behaviour of claimants. This might help to further develop the model and assist its applicability in education for physicians (i.e. the communication skills training course that we are developing for social insurance physicians).

Medical disability assessments are sometimes criticised by Dutch society for not taking the unique disabilities of particular claimants into account. These critiques are best illustrated by remarks from claimants in our prior questionnaire study among 63 claimants [16]. One claimant, for example, said that "she [the social insurance physician] seemed to observe only information that supported her preconceived notions" and another claimant noted: "The physician clearly had his judgement ready, which contradicted the judgement of my occupational physician, internist, and therapist". Of course, these quotes represent the view of the claimant, which may differ from 'reality', and these situations may not occur very often, but this has never been studied.

Therefore, the aim of this paper was to investigate: (1) the content of stereotypes used to classify claimants with regard to the way in which they communicate; (2) the origins of such stereotypes; (3) the advantages and disadvantages of stereotyping in assessment interviews; and (4) how social insurance physicians minimise the undesirable influences of negative stereotyping.

## Methods

### Data-collection and subjects

Data were collected in focus group meetings planned during the regular monthly meetings of groups of social insurance physicians. These groups were recruited by randomly approaching chairpersons from the list of all chairpersons of the monthly meetings of the Dutch Institute of Employee Benefit Schemes (the most important employer of social insurance physicians in the Netherlands). These chairpersons were asked to participate voluntarily with their complete group. All participants had to have been recently involved in face-to-face contact with claimants in a medical disability assessment interview (Table 1 provides more information about Dutch social insurance physicians). The participants agreed to devote one of their meetings to a discussion about their perception of claimants in face-to-face physician-claimant encounters during medical disability assessment interviews, mostly because they considered it to be an important and interesting subject, or because they did not yet came up with another subject for their next monthly meeting. Data were collected in three focus group meetings, which were the first three groups of physicians that agreed to participate in the study within a reasonable time. We declined four other groups that applied, because their availability did not match our time schedule. Also, in two groups not all physicians wanted to participate and thus the groups decided not to join. Because over 10 physicians in one meeting might hinder the discussion and interaction (important ingredients for a successful focus group meeting), the three groups were held separately. In the research design we selected focus group meetings, because little is known about stereotyping in medical disability assessment interviews, and we expected the interaction between the participants to provide more information and more in-depth information than individual interviews.

**Table 1:** Characteristics of Dutch social insurance physicians

In the Netherlands, most social insurance physicians are employed by the Dutch Institute of Employee Benefit Schemes. On average, a physician working there interviews 10 claimants - who may have all kinds of disabilities - each week. The medical disability assessments they perform, are mainly based on an assessment interview, which includes an examination. In addition, usually the physicians have information available from the claimant's occupational physician and the treating physician, or they can consult these professionals [33,34]. Most often, after the interview with the social insurance physician, a labour expert examines which jobs the claimant should be able to perform with the medical disabilities as assessed by the social insurance physician [35]. The combination of the findings of both professionals determines whether or not a claimant is eligible for a benefit.

Three researchers were present at each meeting: a process facilitator, an observer and content expert, and a researcher who took notes. Each focus group meeting lasted for approximately two hours, with a short break after one hour. Because of its negative connotation, the researchers refrained from using the term 'stereotyping' during the focus group meetings. At the beginning of the focus group meeting, the participants were informed about the general aim of the project, being to make an inventory of how social insurance physicians apply classifications of claimants during medical disability assessment interviews, and how these classifications might help or hinder them in the physician-claimant communication. After the meeting was over, the researchers explained more about the study and research project to those who showed interest. A summary of the interview protocol is provided in an additional file 1 (Appendix 1 - summary of the interview protocol). No ethical approval was needed according to the Dutch law, because no claimants were included in the study and the physicians were not exposed to any intervention.

Directly after the meeting, all participants completed a short questionnaire about demographic characteristics. Also, they received a summary of the content of the focus group meeting which they were asked to check. They were asked to contact the researchers if they found any errors or omissions.

### Analysis

All the meetings were audio-taped and transcribed. Qualitative analyses of the transcribed focus group meetings, combined with additional notes taken by one of the researchers, were performed in four successive steps, according to the grounded theory [17,18] and the principle of constant comparison [19]. Firstly, in the exploratory phase, free coding was applied to all data, i.e. all text concerning a particular topic was given a matching descriptive code. Secondly, axial coding was applied, i.e. coding aimed at generalisation of the free codes. This is the phase of specification in which themes and sub-themes emerge. Thirdly, selective coding was applied in the reduction phase. The aim of this phase was to elaborate on the core themes and concepts, and to identify relationships between these themes and concepts. In this phase the results can be summarised in a model. Fourthly, all codes were integrated in the integration phase, and the results of the interviews were compared with those in the formulated model. This entire analysis is an open process in which questions can be adapted for future focus group meetings according to the findings and experiences in former meetings, and therefore only one group is insufficient [19]. The results presented below are the final results after completing the entire analysis.

The software package Atlas.ti 5.2 was used to label the transcripts by assigning codes, to order codes, and to visualise relationships according to the four above-mentioned steps. The first author performed all the coding and the third author also independently performed half of the coding. After all the coding had been completed, a consensus meeting was held. If there were any differences of opinion, the original data were reconsidered until consensus about codes and relationships was established. The data-collection and analysis continued until saturation of information was established, e.g. the transcripts of the meetings provided no new information. Three focus group meetings were enough to achieve saturation.

## Results

### Participants

A total of 22 social insurance physicians participated in the three focus group meetings. The focus groups consisted of eight, six, and eight physicians, respectively. Their mean age was 47 years and 9 months (SD = 7 years and 8 months), on average they had been working as a social insurance physician for 14 years and 2 months (SD = 6 years and 2 months), 14 were male and 8 were female. All the participants currently held medical disability assessment interviews, which was a prerequisite for participation.

Only one group reacted to the content of the summary provided for them to check. In their comments they stressed the importance of certain issues and opinions, and asked for some remarks to be clarified. Their comments were taken into account in the results.

### The content of stereotypes

After generalizing the responses of the physicians to a still higher level of abstraction (deduction to fewer categories), two dimensions on which physicians classify claimants finally remained. Firstly, a dimension concerning the physician-claimant relationship was identified from the combined responses of the physicians. The physicians indicated that they consider the communication of the claimants to be pleasant if they provide clear information, keep a low profile (i.e. do not argue with the physician, show no hostile behaviour), and the assessment takes very little time. This indicates a relationship of respect and acceptance between the physician and the claimant.

"Open claimants, people without a hidden agenda - who say I feel this, I can or can't do that - with that person you think 'this is true', you don't have to ask yourself: is this correct, is this consistent or not? People like that." (male, 50 years old, social insurance physician for 17 years)

Respecting, accepting claimant behaviour is on the one end of the relationship dimension. On the other end, there are claimants who show a lack of respect for the physician and do not accept the physician's role and position. Secondly, a dimension concerning the claimant's influence on the interview was identified. This dimension comprises of dominating and controlling claimant behaviour in the communication during the assessment interview on the one end, and obedient and compliant behaviour on the other end.

Examining these two dimensions, we found that the content of the dimensions bared resemblance to the content of the two orthogonal axes of the interpersonal circumplex (a model for conceptualising and assessing interpersonal behaviour, also known as the Leary circle), because the one dimension concerned solidarity, friendliness, and warmth, and the other dimension concerned status, power, and control. In the literature, different authors name the dimensions on these two axes differently [20-22]. We chose the naming that most closely resembled our findings and is the most appropriate in the context of disability assessments. Thus, we described the dimension on the horizontal axis of our circumplex as running from critical to respecting/accepting and the dimension on the vertical axis as running from dominating to submissive.

**Figure 1:**
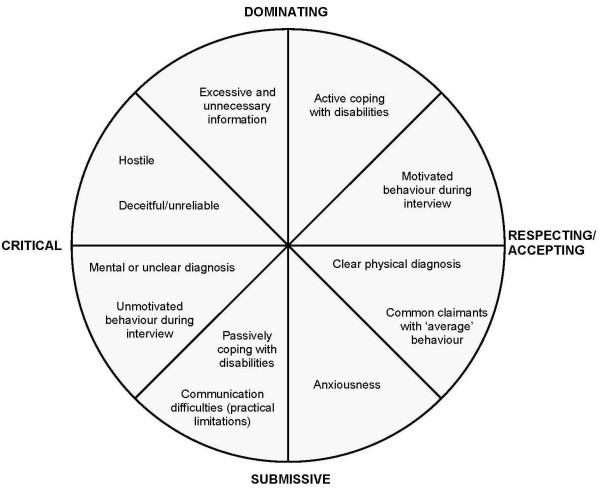
**A typology of claimants reported by physicians which forms the basis for stereotypes based on the interpersonal circumplex**. (more details of each of the categories are provided in Table 2)

Next, we placed our findings within the circumplex, resulting in a communication behaviour typology of eight octants that best matches the physician responses. The two dimensions in the interpersonal circumplex and the typology were fine-tuned and validated by looking (again) at the findings of the individual focus group meetings (following the repetitive process of analysis according to the grounded theory and principle of constant comparison). The typology is presented in Figure 1, and more details are provided in Table 2.

**Table 2:** Categories of claimants reported by physicians which form the basis for stereotypes and their characteristics

Category	Characteristics
Active coping with disabilities	- Remain active - Problem-solving ability - Take responsibility - Take control of their lives - Have adequate introspection - Search for opportunities to continue working or return to work - Think in possibilities - Justify the claims - Possible serious disabilities

Motivated behaviour during interview	- Open - Honest - Straightforward - Willing to co-operate - Claim the disabilities they really have - Accept physician's conclusions

Clear physical diagnosis	- Unambiguous physical disability - Easily understandable disability

Common claimant with 'average' behaviour	- "Just normal claimants" - Rather relaxed - Say things the way they are

Anxiousness	- Tense before interview - Tense during interview - Lack self-confidence - Insecure - Dependent - Uncommunicative

Passive coping with disabilities	- Negative or passive attitude - Lack motivation - External locus of control with regard to coping with their disabilities and continuing work or returning to work - See problems everywhere - Focus on what they can not do - Stress the negative - Suffer from their disabilities - Do not want to work - Feel that they are a victim - Possibly the result of a different cultural background

Communication difficulties (practical limitations)	- Hearing problems - Difficulties with speaking and understanding Dutch - Low level of intelligence - Intellectual disabilities

Mental or unclear diagnosis	- Psychiatric disorders - Personality disorders - Non-specific disorders - Disorders that are difficult to objectify and have an unclear cause (e.g. somatisation, chronic fatigue) - Claim many different disabilities and medical complaints - Inconsistent disabilities - Physical claim, but mental disabilities

Unmotivated behaviour during interview	- Uncommunicative - Elusive - Silent - Passive and uninformed - Dependent - Claim many disabilities - Unwilling to co-operate - Do not say anything spontaneously

Hostile	- Look for confrontations - Intimidating - Threatening - Aggressive (verbally or physically) - Put physician in inferior position - Dominate interview (verbally or physically) - Might "explode" when disagreeing

Deceitful/unreliable	- Deliberately deceitful - Unreliable - Stubborn - Invent disabilities - Have a hidden agenda - Manipulate - Give contradictory and inconsistent information - Might also be "too nice"

Excessive and unnecessary information	- Give an overload of information - Keep talking (physician does not get a chance to intervene) - Autonomous - Elaborate - Pay a lot of attention to relevant as well as irrelevant details - Immediately place all their points on the agenda - Keep changing the subject - Need structure - May exaggerate disability claim in order to justify it

On the 'mutual respect and acceptance' side of the relationship dimension (the half on the right side of the circle in Figure 1), four claimant characteristics are located: actively coping with disabilities, motivated behaviour during the interview, a clear physical diagnosis ("When it's a piece of cake, the physical complaint is just a knee complaint, without much mental fuzz. However, you always have to be open minded because it could be more than just a physical complaint, just a painful knee"), and anxiousness. The physicians also stated that the majority of the claimants they meet are 'common' claimants with no 'striking' characteristics and with 'average' behaviour, and that they usually establish a relationship of respect and acceptance with such claimants.

The opposite side of the relationship dimension (i.e. a relationship based on other things than respect and acceptance) contains opposite characteristics: passively coping with disabilities, unmotivated behaviour during the interview, and a mental or unclear diagnosis. Communication problems (e.g. hearing problems, intellectual disabilities) can also be found there. On the dimension of the claimant's influence on the interview, these characteristics are all on the more 'submissive' side (the lower left quadrant in Figure 1): claimants take a submissive position in interacting with the physician. These claimants make the interview time-consuming or rather difficult.

One physician characterised claimants who passively cope with their disabilities as:

"The person who sees problems everywhere. Who thinks of 10 problems for every solution you suggest. Also, 10 solutions to every problem but, according to them, they are all no good." (female, 34 years old, social insurance physician for 5 years)

Another physician confirmed the problems of lack of motivation in the interview:

"They don't know, so they go along completely with my story, but that's not what I want. I want information, but that's not what I get. When I facilitate the conversation, I just fill in the blanks according to my own ideas, but I already know those. I'm interested in what *they *do, but they don't say anything. They give you the feeling that, no matter how hard you work, you will never get where you want to be. And then you work really hard, but that doesn't help either." (male, 51 years old, social insurance physician for 22 years)

The physicians stated that passively coping with disabilities might be due to a different cultural background, because in the Dutch social security benefits system a person is held responsible for his/her own behaviour and its consequences. They argued that people with a different cultural background take one day at a time, do not take personal responsibility, and are not expected to have any control over their life. This creates barriers "because you try speaking in Dutch, or you try to explain the consequences of the Dutch law to such a person, but they can't understand, because it doesn't fit in with their culture". The physicians found it difficult to asses claimants with a mental diagnosis or an unclear 'physical' diagnosis:

"You actively have to search for what exactly is going on. Of course, we're talking about those syndromes for which it has already been said that they're vague, they're non-specific. Certainly, with those syndromes I'm always suspicious, and wonder what else could be the matter?" (male, 48 years old, social insurance physician for 27 years)

The physicians also mentioned characteristics that classify the physician-claimant relationship as lacking in respect and acceptance, combined with a dominating attitude that has considerable influence on the interview (the upper left quadrant in Figure 1). This group includes claimants who are inclined to provide excessive and unnecessary information ("And it is not exactly that they won't co-operate, but you've lost control over the interview. And that makes the interviews longer than you'd intended") or whose behaviour is hostile, deceitful and/or unreliable ("Right, that man had arms that were bigger than my whole body, so to speak, so I think if he had hit me ... He was so full of anger, facing me. I thought, be careful now").

The physicians reported that they deliberately adapt their communication style to the claimant's style of behaviour (and thus to their stereotype of the claimant, as summarised in the four quadrants of the typology). For example, in interviews with claimants with dominant communication behaviour and a lack of respect in the relationship, physicians take care not to end up in an inferior position, they are cautious in their decision-making (because information might be missing or is not correct), they ask more in-depth questions, and they are more alert:

"Then you start questioning them more, about their routine and their daily activities, for example, which reflects their capacity. To check whether their functional complaints match the things they tell me. That's how I try to find out." (male, 51 years old, social insurance physician for 9 years)

### Origins of stereotypes

Most physicians reported that they were retrospectively aware that they unconsciously classify claimants in general groups. They saw this process as a characterisation or arrangement in their heads, a frame of reference, resulting from prior experiences. Based on this frame of reference they adapt their behaviour. However, some physicians stated that they never apply stereotypes: they reported that they behave and communicate in the same way with all claimants, that their first impressions do not influence the interview, and that their reactions are always a direct consequence of what happens in the interview: "Actually, I start the interview in the same way with every person". Nevertheless, focus group discussions revealed that all physicians do make classifications on the first encounter, further on during the interview, and also after the interview. Stereotyping after the interview occurs, in particular, when writing down the findings in the file, thinking back on the interview, and discussing the interview with colleagues. Physicians deal with stereotypes both consciously (deliberately) and unconsciously.

The physicians reported that the opinion about a claimant on the first encounter is based both on the information in the file and the first impressions when meeting the claimant in person. Physicians compare the information in the file with their memories of other, similar claimants, and then see a pattern: "Of course you create an image for yourself. For example, when I read that the interview will be with a 32 year-old teacher, I've already got a complete mental image, because I've already seen 500 of them". In addition, the medical anamnesis and the reports written by other social insurance physicians who previously met the claimant often paint a clear picture: "I think there's a difference between seeing a person for the first time and having a complete file with information from several social insurance physicians who have seen that person before". Combining this information gives rise to expectations, opinions, feelings, and biases about the claimant.

Subsequently, when the physician meets the claimant for the first time, the sight of the person in the waiting room, their way of shaking hands, and other non-verbal signals also influence the physician's impression of the claimant. The physicians stated that these first impressions are useful, because they only have approximately half a minute to decide on how to approach the claimant. Furthermore, they also use first impressions "as a diagnostic tool. If you think that someone is compulsive or manic - for example people who won't stop talking - you ask other questions to test that presumption".

### Advantages and disadvantages of stereotyping

Although stereotyping has its disadvantages, according to the social insurance physicians in the focus group meetings, the information it provides can also be useful. For the physicians the main advantages of having a mental picture of what claimants will be like, before meeting them, were: (1) it provides a framework for the assessment interview; (2) it can save time; and (3) it is interesting to check whether the classification is correct.

Firstly, a practical advantage was that stereotypes provide a framework for the assessment interview, which means that the physician can prepare more thoroughly and has less reason to feel insecure: "I want to prepare well, I want to be able to assess to some degree what I might run into. And that people know that I have prepared". The physician can anticipate the effort that must be made to gather information, the eagerness of the claimant to oppose or to irritate the physician (including possible hidden agendas), and the likelihood that the claimant will file a complaint. Moreover, stereotypes provide the physician with a theory to test the claimant and the claimant's disabilities, and the physician can use the stereotype for diagnostic purposes.

Secondly, stereotyping has the practical advantage that it can help to save time. All the physicians thought that this was important:

"Saving time is important given our circumstances ... We need a lot of information in a short time. We run into time limitations." (female, 41 years old, social insurance physician for 14 years)

Stereotyping claimants can shorten the interview, because the focus of the interview can be determined beforehand, and more effective preparation saves time. For example, collecting information about the disability of the claimant can accelerate the interview and prevent unnecessary sidetracking, and inferences concerning the cultural background of claimants may increase understanding of their disabilities: "The ultimate goal is gathering information within an hour. And than, you have to - with the help of the techniques you know - get that information clear. And depending on the different groups you will have to adjust". However, when physicians classify the claimant wrongly, the interview will probably take more time, instead of less.

Thirdly, some physicians argued that it is rewarding to find out whether their stereotypes are correct. They form an opinion of the claimant, and test this hypothesis for its accuracy: "A little 'professional curiosity' ... I can amuse myself with that". Usually, the stereotype is confirmed or rejected. Especially when the reality is exactly the opposite of the expectations, this can motivate the physician to be more cautious and accurate next time, and keeps it interesting. One of the physicians explained this as follows: "Beforehand you create an image, and sometimes also real prejudices ... Then I enjoy being confronted with these, and I think: it's going to be a difficult interview ... Then afterwards I could have kicked myself and my prejudices, nothing about a human being is foreign to me. Yeah, that's fun".

As stated before, stereotypes often prove to be incorrect, and expectations often remain unmet. This is one of the disadvantages of stereotyping that was mentioned by the physicians. The two other disadvantages they mentioned, are: a stereotype does not give the complete picture, and because people are dynamic constant adjustment is needed anyway.

Firstly, the fact that stereotypes often prove to be incorrect and expectations often remain unmet is illustrated by these citations: "At the same time, that's the weakness, because you never know" and "You think: oh, it will be one of those people. At that moment ... it's quite different from what you had expected". The physicians emphasised that it is important to stay as free from value judgements as possible. This is also to prevent unnecessary worrying beforehand, and to prevent an unpleasant atmosphere during the interview. Moreover, stereotyping might cause the physician to miss certain information.

Secondly, the physicians argued that a stereotype does not give them the complete picture; there is much more that should be taken into account, and "classifying in types is one aspect, but you can't base an entire interview on that". The situation (e.g. why a claimant is on sick leave), environment, social network, and intelligence of the claimant are also important, just like the physician's characteristics and the dynamics of the physician-claimant contact. Moreover, the moment at which the interview takes place is also important: "And that defines standards and values. Then you can have a person with many substantial symptoms of rheumatism and several adaptations, and he's willing to work, and another person who barely has any disability and ... then you think 'what a whiner' - but you don't say it - compared to the other person [with many substantial disabilities]. Things like that do interfere with medical decision-making." Furthermore, not every claimant will fit into a classification, or match a stereotype, and many complex claimants are "nondescript figures" (i.e. average, unnoticed people with very few pronounced stereotypes).

Thirdly, an important disadvantage of stereotyping is that claimants are dynamic, and therefore physicians have to constantly make adjustments during the interview. Classification in stereotypes is stable, whereas the reality of an interview is an ever-changing dynamic process, and thus, as this physician concisely formulated:

"Interviews from the past don't give guarantees for the future." (female, 61 years old, social insurance physician for 15 years)

Moreover, the classification of a claimant might vary considerably during an assessment interview, for example depending on the phase of the interview (i.e. the claimant can be co-operative in giving information, but not co-operative when informed about decisions). Therefore, stereotypes have to be adjusted continuously.

### Minimising undesirable influences of stereotypes

The physicians agreed that stereotypes are often unproductive or undesirable, and therefore the negative influence of stereotyping should be minimised. They used several strategies to achieve this aim: (1) being aware of counter transference; (2) making very formal assessments; (3) staying neutral to the best of their ability; and (4) being compassionate.

Firstly, being aware of counter transference means that the physician is aware of his or her biases and prejudices with regard to claimants: "That gives rise to a particular prejudice, which is okay, but you need to be aware of it". During the assessment interview the physicians show this awareness by discussing findings and opinions with the claimant openly. This implies that "if you've trouble dealing with a particular type of patient, you should first take a look at yourself, because you're the only one who knows what bothers you. Your personality determines your allergies". Outside the interview, discussing stereotypes with colleagues in discussion groups, supervision, or even psychotherapy is recommended:

"We also confer with each other, we talk about things and hear from each other ... That also has to do with your own perception: your own attitude to life and what you expect." (male, 42 years old, social insurance physician for 9 years)

Many of the physicians argued that, when they know that they have a stereotype image of a claimant, they are able to 'un-stereotype' just as easily as they stereotyped, although some said "but very often you just continue with your first impression". When aware and unable to eliminate all influences, they might also consult other sources of information, for example medical specialists who are treating the claimant.

Secondly, the assessment is made in a formal way, according to a structured assessment method, specifically focusing on the information that is needed, or by applying a structured conversation/communication technique. The physicians try to create a clear structure for the claimant, they are directive, they take their time to gather all the necessary information, they try to make contact in such a way that they obtain the most information from the claimant (e.g. "And there are different ways to treat people, depending on their abilities, their needs, what they don't want, what they do want, their motivation, their intentions, and so on."), and they adapt to the claimant's intellectual level of conversation (e.g. using easier wording and language). They try not to become irritated, or to put pressure on themselves. When necessary, claimants are asked to write down their opinions and concerns in a letter that will be added to their file.

Thirdly, the physicians stay neutral by telling themselves to start with an unbiased, open-minded, objective attitude, and to be free-and-easy in the interview, also trying to avoid value judgements: "Then I have that all in mind and then I say to myself, no, go into the consulting room with a neutral, unbiased attitude.". The physicians stated that they listen to claimants, take them seriously, and first follow their line of reasoning and let them tell their complete story before asking more in-depth questions. They try to readjust during the interview if they notice that the influence of a stereotype increases:

"At first you're neutral, but at a certain moment you adapt your interviewing technique to the person, to the person's intellect, to the person's reactions, because in the end your goal is to gather information within an hour. And with your techniques, you have to uncover that information. And depending on different groups you have to adapt." (male, 44 years old, social insurance physician for 18 years)

Fourthly, the physicians indicated that they are compassionate. They openly discuss the claimant's findings, opinions, and impressions with the claimant, and they mirror the claimant's behaviour. One social insurance physician said that she acts in the opposite way to the claimant to elicit different behaviour (e.g. being very cheerful with a depressed claimant). Moreover, they also mentioned showing respect and sincere interest, comforting claimants, letting claimants know that they understand them, and taking a positive attitude. That is what it is all about: "Our profession actually has more to do with social contact. It's not about being formal. We try to communicate in such a way that people feel at ease when they tell their story".

## Discussion

### Main findings

Qualitative analysis of the focus group meetings with the social insurance physicians showed that claimant behaviour can be categorised into the following dimensions: 'respect and acceptance in the physician-claimant relationship' and 'the claimant's influence on the interview'. Combined, these dimensions resulted in a communication behaviour typology with eight octants with regard to the communication during assessment interviews. Physicians adapt their communication style to the claimant, depending on the location of the claimant's behaviour on both dimensions. Although stereotyping is usually an unconscious process, the physicians were aware that it was happening. They explained this as a frame of reference, resulting from prior experiences. Stereotypes mainly result from first impressions when reading the file and the first actual encounter. The physicians were of the opinion that stereotyping has advantages and disadvantages. The main advantages were: it provides a framework for the assessment interview, it can save time, and it is fun to check whether the classification is correct. However, they also thought that there are several important disadvantages: stereotypes often prove to be incorrect and expectations often remain unmet, a stereotype does not provide the physician with the complete picture, and because people are dynamic you constantly have to make adjustments. Therefore, to minimise the negative influence of stereotyping, physicians apply four strategies: being aware of counter transference, making a very formal assessment, staying neutral to the best of their ability, and being compassionate.

### Findings in relation to other studies

Our aim was to investigate whether, and if so, how stereotyping might influence medical disability assessments. Although the literature shows that objectivity in this respect is an illusion [10,11], some physicians stated that they are not influenced by stereotypes. Nevertheless, their responses during the focus group meetings did indicate that they did apply stereotypes. Studies have convincingly shown that awareness of stereotypes and the motivation not to apply stereotypes is not enough to prevent their influence, but awareness and motivation are helpful [23]. Thus, teaching physicians who lack awareness - and therefore motivation - about stereotypes is an important challenge for future intervention studies [23]. Findings reported in the literature, that stereotyping might influence the interpretation of symptoms and behaviour [7], are in line with our findings that symptoms and behaviour are characteristics according to which claimants are classified (i.e. clear physical diagnosis, mental or unclear diagnosis, respectively coping behaviour, behaviour during the interview). In general, the literature suggests that the motivation of claimants [5,9] is a relevant characteristic for physicians who make medical disability assessments, and their communication styles [7] did, indeed, seem to be affected by the stereotypes. Physicians indicated that they adjusted their communication to the behavioural style of the claimant, and this style seemed to be determined by stereotyping, among other things.

The results of our study replicated several general findings in medical disability assessment interviews: that physicians apply stereotypes and this increases their comprehension of patient behaviour [7,10], that physicians experience problems with stereotyping, and that they prefer individual information, and therefore try to minimise the influence of unproductive stereotypes [2]. With regard to the content of stereotypes, our results are also in line with reports in the literature. As mentioned before, the results can be placed in the interpersonal circumplex [20,21]. Moreover, the behaviour of the physicians towards the behaviour of the claimants is consistent with the predictions of the circumplex [24]: a respectful relationship initiated by the claimant evokes respectful behaviour from the physician; disrespectful behaviour evokes disrespectful behaviour, and a submissive claimant evokes an active, dominating response from the physician. However, a dominant claimant does not evoke a submissive response from the physician, which might be because physicians are extra alert with this type of claimant and take care not to end up in an inferior position. Moreover, Balsa and McGuire [25] showed that the patient's degree of co-operation and the physician's degree of effort both influence the physician's stereotyping with regard to patient behaviour. Our results concerning the dimension of mutual respect and acceptance, reflect this degree of co-operation, and our finding that whether or not claimants show a critical, dominating attitude is important for physicians, reflects this degree of effort. Examples of both 'automatic stereotyping' and 'goal-modified stereotyping' [7] were found.

It is known that stereotyping depends on the social context [10,14]. Our results did not support the importance of general social characteristics, such as age and gender, in stereotyping by social insurance physicians, but the physicians did indicate that they consider the type of disability of the claimant (i.e. physical or psychological complaints) and the claimant's way of coping with disabilities to be important in determining their method of communication. These categories are quite relevant and salient in medical disability assessments, and therefore easily linked to stereotyping [14]. The physicians stated that the cultural background of claimants is a relevant category for classification. This finding is noteworthy, because cultural stereotypes may lead to perceiving people originating from the same cultural background as physically and culturally uniform [4], and subsequently different care for different groups of people (e.g. ethnic disparities) [23]. In addition, there is a risk of 'self-stereotyping', that is: claimants evaluate themselves more in line with a negative stereotype when they belief that a person with power over them holds that stereotypic view [26]. Both consequences of stereotyping regarding cultural background might influence the result of the medical disability assessment.

Three goals for stereotyping are generally distinguished in the literature: self-enhancement goals, comprehension goals, and motivation to avoid prejudice [2,7], and these are reflected in our findings. Firstly, self-enhancement goals correspond with the finding that physicians' classify claimants according to the degree of positivity of the physician-claimant relationship. Labelling a claimant as 'negative' or 'critical' might be a reason for communication problems or difficulty in drawing the correct conclusions. Secondly, the physicians mentioned comprehension goals, in that stereotypes provide a framework for the assessment and can make preparation for the interview more effective. However, they also indicated that comprehension could be hindered by stereotypes if it does not provide the complete picture. Thirdly, the physicians were motivated to avoid prejudice, because they found it interesting to check whether the stereotypes were correct, and also mentioned the disadvantages of stereotyping. Our findings therefore seem to be in agreement with the 'goal-based framework for stereotype activation and application' according to Kunda and Spencer [2]. In their framework, self-enhancement goals and comprehension goals, together with stereotype activation, stimulate stereotype application, and simultaneously, the motivation to avoid prejudice inhibits stereotype application.

Several concepts in our previously published theoretical model [15] match the findings from the current study. For example, we conceptualised a passive coping attitude, a wait-and-see coping attitude, and an active coping attitude, which correspond to the dimension of a submissive (first two) versus dominating (third) claimant in the typology. Similarly, the dimension of critical versus respecting/accepting relationship in the typology corresponds to the conceptualisation of a result-directed attitude versus a relationship-focussed attitude. The other attitudes in our framework: the attitude regarding patient-centeredness and the attitude about expression of emotions, also match the findings, but more indirectly. These are included in characteristics such as hostility and anxiousness. Overall, the typology seems to confirm the main concepts of the theoretical framework.

### Strengths and limitations of this study

This study has several strengths, as well as some limitations. The strengths are: (1) the data-analysis procedure, (2) the participants, and (3) the environment in which the focus group meetings were held. Firstly, although the data were qualitative and not quantitative, they were processed and analysed in a systematic and structured way. Secondly, the participants in the focus group meetings had many years of experience as social insurance physicians. Thirdly, the focus group meetings took place in a familiar and safe environment, in which the physicians had already had the opportunity for self-reflection, talking about sensitive issues, speaking freely, and open discussions. This made the discussions easier, and it was therefore less likely that their answers and opinions would be socially desirable.

Limitations of this study are: (1) the controversy of using stereotypes in relation to the method of data-collection; and (2) unconscious stereotyping was studied by asking participants about their conscious awareness. Firstly, stereotyping appears to be a taboo among social insurance physicians, even though it has been shown that it is valid to differentiate between patients on the basis of characteristics such as age, social circumstances, and gender [27]. The controversy of stereotyping could cause a problem, because we relied on verbal reports from the participants, which implies that they might under-report their application of stereotypes. Secondly, there is a contradiction in asking people about an unconscious process. The social insurance physicians were probably neither aware of their stereotyping behaviour nor the stereotypes they apply. We tried to minimise these limitations by asking indirect and general questions (instead of only personal questions), and by asking the physicians to give examples.

Within this study no time remained to validate the results, particularly the typology, in another way than by asking the physicians about their opinions in the focus groups. However, it would be interesting to use in depth interviews or a quantitative study to further validate these findings and this typology.

### Implications for practice

The physicians indicated that there are both disadvantages and advantages of stereotyping, and because of the possible negative consequences, they try to be aware of the processes of stereotyping and try to minimise the undesirable influence of stereotyping. Their strategies to avoid counter transference and to discuss prejudices about claimants with colleagues are useful in this respect [28,29], but paying explicit attention to being compassionate might also be important. These strategies could be taught in training courses or other educational settings for less experienced physicians, or to increase awareness of the potential influence of stereotyping in general. Since medical decisions, and thus also medical disability assessments, depend on clinical reasoning [30], awareness of the potential influence of stereotyping is important. Moreover, because it is known that a decrease in cognitive capacity can increase reliance on stereotypes and stereotype-confirming information [10], attention should be paid to the time limitations and information overload (and the fatigue that could result from this) that some social insurance physicians experience.

One could argue that there is a tension between the process of observing claimants' behaviour for determining their work capacity and that of observing behaviour to form a stereotype. In determining work capacity, physicians have to recognise a pattern, find evidence to confirm this pattern, and thereby make a diagnosis [12]. Similarly, in stereotyping physicians recognise a pattern in claimant behaviour. The tension between these two processes comes from the notion that the first process of stereotyping is acceptable, but the last process is unwanted and only has disadvantages. However, this notion is not defensible because, firstly, the physicians in the focus group meetings indicated that they sometimes use stereotypes as a diagnostic tool. Secondly, stereotypes are needed to comprehend others and also have other advantages (as our study showed). It is nevertheless important - because both diagnosing and stereotyping include generalisation - that physicians carefully check to what degree the pattern or stereotype matches the individual claimant and what specific additional individual information is needed.

Our results showed that social insurance physicians adjust their communication to the degree of respect in their relationship with the claimant. With respectful claimants, an instrumental communication style, paying little attention to the possible empathic, affective needs of claimants is usually sufficient, and therefore compassion is predominantly reserved for interviews with 'critical' claimants. Because it is known from the literature that empathy influences the diagnosis, patient satisfaction, coping with bad news, and adherence to medical recommendations [31,32], this is an important finding that should be incorporated in future training courses. Training physicians to apply the interpersonal circumplex to medical disability assessments might be beneficial in this respect. It is therefore important to address the awareness and handling of stereotypes in education and training for social insurance physicians.

## Conclusions

Physicians are partly aware of the influences stereotypes might have on their communication with claimants and on their decision-making. During assessment interviews, physicians adapt their communication style to the degree of respect and dominance in the claimant's communication. This increases their comprehension of the way in which claimants communicate. Simultaneously, physicians often prefer to receive individual information, which is more accurate, and therefore try to minimise the negative influences of stereotyping on the interviews. Communication skills training or other training courses for physicians should focus on increasing awareness of the influences of stereotyping, by discussing stereotypes and prejudices. The most effective ways to minimise the undesirable influences of stereotyping should also be addressed.

## Competing interests

The authors declare that they have no competing interests.

## Authors' contributions

HJvR and AJMS came up with the idea for the manuscript. HJvR wrote most of the manuscript and MB wrote some parts of the manuscript. AJMS, JRA and AJvdB revised and commented on the manuscript. All authors have read and approved the final version of the manuscript.

## Pre-publication history

The pre-publication history for this paper can be accessed here:

http://www.biomedcentral.com/1471-2458/10/666/prepub

## Supplementary Material

Additional file 1 Summary of the interview protocol Click here for file

## References

[B1] Ames DR Strategies for social inference: a similarity contingency model of projection and stereotyping in attribute prevalence estimates J Pers Soc Psychol 2004 87 573 585 10.1037/0022-3514.87.5.573 15535772

[B2] Kunda Z Spencer SJ When do stereotypes come to mind and when do they color judgment? A goal-based theoretical framework for stereotype activation and application Psychol Bull 2003 129 522 544 10.1037/0033-2909.129.4.522 12848219

[B3] Van den Bos A Stapel DA Why people stereotype affects how they stereotype: the differential influence of comprehension goals and self-enhancement goals on stereotyping Pers Soc Psychol Bull 2009 35 101 113 10.1177/0146167208325773 19106080

[B4] Alter AL Darley JM When the association between appearance and outcome contaminates social judgment: a bidirectional model linking group homogeneity and collective treatment J Pers Soc Psychol 2009 97 776 795 10.1037/a0016957 19857001

[B5] Van Brakel WH Measuring health-related stigma - a literature review Psychol Health Med 2006 11 307 334 10.1080/13548500600595160 17130068

[B6] Butz DA Plant EA Prejudice control and interracial relations: the role of motivation to respond without prejudice J Pers 2009 77 1311 1341 10.1111/j.1467-6494.2009.00583.x 19686455

[B7] Burgess DJ Van Ryn M Crowley-Matoka M Malat J Understanding the provider contribution to race/ethnicity disparities in pain treatment: insights from dual process models of stereotyping Pain Med 2006 7 119 134 10.1111/j.1526-4637.2006.00105.x 16634725

[B8] Bos AE Schaalma HP Pryor JB Reducing AIDS-related stigma in developing countries: the importance of theory- and evidence-based interventions Psychol Health Med 2008 13 450 460 10.1080/13548500701687171 18825583

[B9] Dodor EA Kelly S Neal K Health professionals as stigmatisers of tuberculosis: insights from community members and patients with TB in an urban district in Ghana Psychol Health Med 2009 14 301 310 10.1080/13548500902730127 19444708

[B10] Burgess DJ Fu SS Van Ryn M Why do providers contribute to disparities and what can be done about it? J Gen Intern Med 2004 19 1154 1159 10.1111/j.1525-1497.2004.30227.x 15566446 PMC1494785

[B11] Ryan CS Robinson DR Hausmann LR Stereotyping among providers and consumers of public mental health services. The role of perceived group variability Behav Modif 2001 25 406 442 10.1177/0145445501253003 11428247

[B12] Bonilauri Ferreira AP Ferreira RF Rajgor D Shah J Menezes A Pietrobon R Clinical reasoning in the real world is mediated by bounded rationality: implications for diagnostic clinical practice guidelines PLoS One 2010 5 e10265 10.1371/journal.pone.0010265 20421920 PMC2857648

[B13] Croskerry P A universal model of diagnostic reasoning Acad Med 2009 84 1022 1028 10.1097/ACM.0b013e3181ace703 19638766

[B14] Link BG Phelan JC Conceptualizing stigma Annu Rev Sociol 2001 27 363 385 10.1146/annurev.soc.27.1.363

[B15] Van Rijssen HJ Schellart AJ Anema JR Van der Beek AJ A theoretical framework to describe communication processes during medical disability assessment interviews BMC Public Health 2009 9 375 10.1186/1471-2458-9-375 19807905 PMC2765440

[B16] Van Rijssen HJ Schellart AJ Anema JR Van der Beek AJ A typology of sick-listed claimants to improve communication skills for social insurance physicians during medical disability assessment interviews J Occup Rehabil 2010 in press 20623165 10.1007/s10926-010-9254-4

[B17] Glaser BG Strauss AL The discovery of grounded theory: strategies for qualitative research 1967 Chicago: Aldine

[B18] Strauss AL Corbin J Basics of qualitative research: techniques and procedures for developing grounded theory 1998 Thousand Oaks: Sage

[B19] Boeije HR Towards a purposeful approach of the constant comparative method in the analysis of qualitative interviews Quality & Quantity 2002 36 391 409 10.1023/A:1020909529486

[B20] Freedman MB Leary TF Ossorio AG Coffey HS The interpersonal dimension of personality J Pers 1951 20 143 161 10.1111/j.1467-6494.1951.tb01518.x 14918048

[B21] Leary T Commentary J Pers Assess 1996 66 301 307 10.1207/s15327752jpa6602_8 16367702

[B22] Wiggins JS An informal history of the interpersonal circumplex tradition J Pers Assess 1996 66 217 233 10.1207/s15327752jpa6602_2 16367700

[B23] Van Ryn M Fu SS Paved with good intentions: do public health and human service providers contribute to racial/ethnic disparities in health? Am J Public Health 2003 93 248 255 10.2105/AJPH.93.2.248 12554578 PMC1447725

[B24] Kiesler DJ Auerbach SM Integrating measurement of control and affiliation in studies of physician-patient interaction: the interpersonal circumplex Soc Sci Med 2003 57 1707 1722 10.1016/S0277-9536(02)00558-0 12948579

[B25] Balsa AI McGuire TG Prejudice, clinical uncertainty and stereotyping as sources of health disparities J Health Econ 2003 22 89 116 10.1016/S0167-6296(02)00098-X 12564719

[B26] Sinclair S Pappas J Lun J The interpersonal basis of stereotype-relevant self-views J Pers 2009 77 1343 1364 10.1111/j.1467-6494.2009.00584.x 19686454

[B27] Safran DG Rogers WH Tarlov AR McHorney CA Ware JE Jr Gender differences in medical treatment: the case of physician-prescribed activity restrictions Soc Sci Med 1997 45 711 722 10.1016/S0277-9536(96)00405-4 9226794

[B28] Beagan BL Kumas-Tan Z Approaches to diversity in family medicine: "I have always tried to be colour blind" Can Fam Physician 2009 55 e21 e28 19675253 PMC2726109

[B29] Heijnders M Van der Meij S The fight against stigma: an overview of stigma-reduction strategies and interventions Psychol Health Med 2006 11 353 363 10.1080/13548500600595327 17130071

[B30] Montgomery K Thinking about thinking: implications for patient safety Healthc Q 2009 12 Spec No Patient e191 e194 19667768 10.12927/hcq.2009.20948

[B31] Halpern J What is clinical empathy? J Gen Intern Med 2003 18 670 674 10.1046/j.1525-1497.2003.21017.x 12911651 PMC1494899

[B32] Stepien KA Baernstein A Educating for empathy. A review J Gen Intern Med 2006 21 524 530 10.1111/j.1525-1497.2006.00443.x 16704404 PMC1484804

[B33] De Boer WEL Besseling JJM Willems JHBM Organisation of disability evaluation in 15 countries Prat Organ Soins 2007 38 205 217 http://www.doaj.org/doaj?func=abstract&id=240001

[B34] Spanjer J Krol B Popping R Groothoff JW Brouwer S Disability assessment interview: the role of detailed information on functioning in addition to medical history-taking J Rehabil Med 2009 41 267 272 10.2340/16501977-0323 19247547

[B35] Spanjer J Krol B Brouwer S Groothoff JW Inter-rater reliability in disability assessment based on a semi-structured interview report Disabil Rehabil 2008 30 1885 1890 10.1080/09638280701688185 19037781

