# Supplementary material for: Stereotyping of medical disability claimants' communication behaviour by physicians: towards more focused education for social insurance physicians
Source: BMC Public Health. 2010 Nov 3;10:666. doi: 10.1186/1471-2458-10-666 (PMC2989961; doi:10.1186/1471-2458-10-666)
Supplement: Additional file 1 — Summary of the interview protocol [file 1471-2458-10-666-S1.PDF]

Additional file 1

Appendix 1 – Summary of the interview protocol

Time-schedule

Duration	Subject
10 minutes	Start and introduction
5 minutes	Getting acquainted
40 minutes	Introduction to classification of claimants<br>Why/the goals of classifications<br>How do classifications arise
10 minutes	Break
50 minutes	Verifying classifications<br>Application of classifications in the communication<br>Advantages and disadvantages of classifications
20 minutes	Final questions and closing

Start and introduction

Welcome to the participants.

Introduction to the researchers and the study.

Explanation of the goals and course of the focus group meeting (including comments concerning audio-recordings and confidentiality).

Getting acquainted

Names and nameplates, participants introduce themselves.

Introduction to classification of claimants

Introductory assignment: on three separate memos each participant makes a 'top 3' of types of claimants (with regard to the communication during medical disability assessment interviews) they encounter most frequently. These memos are collected on a large sheet of paper. Agreements, differences, types that are frequently mentioned, and types that are rarely mentioned are discussed.

Questions: Do you or your colleagues classify in types? How often? What are your experiences with using classifications? Why are classifications applicable to claimants and why not? Do you use classifications to facilitate communication?

Why/the goal of classifications

Main question: What are the goals of classification?

Follow-up questions: What are the instrumental goals? What are the affective goals? How are classifications used in the communication with claimants? Do classifications change the way you communicate and behave during disability assessment interviews?

How do classifications arise

Main question: Based on which aspects are claimants classified in groups?

Follow-up questions: Which aspects contribute to the classification of a claimant in a certain group? How does generalisation occur? How do you recognise the type that a claimant belongs to?

Break

Participants are asked not to talk about any subjects discussed in the meeting during the break.

Verifying classifications

Participants are given a summary of the discussions that took place before the break.

Main question: How do you verify classifications?

Follow-up questions: How do you know whether a classification is correct? How do you check the validity of a classification during assessment interviews? How do you adjust a classification if it is incorrect?

Application of classifications in the communication

Main question: How are classifications applied?

Follow-up questions: When a claimant is classified as a type, which information does that provide you with? If you classify a claimant, what is the effect on the communication? If you do not apply classifications, how do you make sure that classifications do not unconsciously influence your way of communicating?

Advantages and disadvantages of classifications

Main question: Which aspects of classifications are found to be helpful in the communication and which are found to be a hindrance?

Follow-up question: How would you be better able to handle aspects that are a hindrance?

Final questions and closing

Summarising and checking all that has been discussed.

Is there anything that should be added?

Participants are thanked for their participation.

Participants fill in the questionnaire.
